# Impact of GI Tumor Board on Patient Management and Adherence to Guidelines

**DOI:** 10.1200/JGO.17.00164

**Published:** 2018-03-23

**Authors:** Haneen A. AlFarhan, Ghada F. Algwaiz, Hajer A. Alzahrani, Roaa S. Alsuhaibani, Ashwaq Alolayan, Nafisa Abdelhafiz, Yosra Ali, Sami Boghdadly, Abdul Rahman Jazieh

**Affiliations:** **Haneen A. AlFarhan**, **Ghada F. Algwaiz**, **Hajer A. Alzahrani**, and **Roaa S. Alsuhaibani**, King Saud bin Abdulaziz University for Health Sciences; **Ashwaq Alolayan**, **Nafisa Abdelhafiz**, **Yosra Ali**, **Sami Boghdadly**, and **Abdul Rahman Jazieh**, King Abdulaziz Medical City, Riyadh, Saudi Arabia.

## Abstract

**Purpose:**

As the burden of cancer on the population and the health care system continues to increase with more complicated treatment options, the need for multidisciplinary teams to be as efficient as possible becomes more vital. Our study aimed to evaluate the consistency of GI Tumor Board (GI TB) recommendations with international guidelines, the adherence of physicians involved in patient care to TB recommendations, and the impact on the management of patients.

**Methods:**

A prospective cohort study was conducted from January to June 2016 at our institution, which is a major tertiary hospital that provides comprehensive cancer care. All cases presented at the GI TB during this period were included. Data regarding adherence to National Comprehensive Cancer Network guidelines, adherence to TB recommendations, and changes made to the management of patients were collected weekly from the GI TB in a data collection form.

**Results:**

Of the 104 patients included, 57 (55%) were males and the median age was 58 (16 to 85) years. Colorectal cancer was the most common diagnosis, in 65 patients (63%). Nearly one-half of cases (45%) were stage IV cancers. Starting new treatment was recommended for 72 patients (69%). Further investigations were requested for 15 patients (14%). For imaging, 24 recommendations (23%) were made. Adherence to National Comprehensive Cancer Network guidelines was observed in 97% of total recommendations. New findings were found in pathology (11%), radiology (13%), and staging (4%). Management plans were changed in 37 cases (36%). Over a 3-month period after presentation to the GI TB, most of the recommendations (87%) were performed.

**Conclusion:**

A multidisciplinary tumor board enhances the adherence to guidelines and has an impact on patient management in approximately one-third of patients. Among physicians, adherence to recommendations of the TB was high.

## INTRODUCTION

Multidisciplinary tumor boards (TBs) are a collaboration of specialists and subspecialists from a variety of fields who gather to review and discuss (general or site-specific) cancer cases. Through this, they can provide a chance to integrate knowledge for possibly a better outcome for the patients.

TBs began as a place to educate and review interesting cases, but in recent years have become a forum with great potential to provide better care to decrease treatment-related morbidity and to improve outcome. In addition to aiding in the decision making of diagnosis and treatment, the importance of TBs also lies in quality improvement, education, and professional career enhancement.^1^

TBs offer physicians the chance to review their cases and to reconsider changes in diagnosis and management. According to one study, the clinical management of 20% of cases presented at the TB was changed as the result of cautious review of patients’ data.^2^ In another study, the management of patients with breast cancer was changed in 11% of cases reviewed during a TB meeting in a surgery department.^3^

The response of clinicians to TB recommendations is as noteworthy as the adjustment of best options for individual patients. Ung et al^4^ demonstrated that implementation of recommendations was completed in 72% of cases. The reasons for not following the recommendations in some cases were pre-existing poor patient performance status, declining performance status, clinician preference, the availability of new data, and patient decision.

As medical practices continue to become increasingly specialized, the need for the multidisciplinary team to be as efficient as possible becomes more vital. To achieve this, TBs have the duty to provide recommendations that represent the best available evidence-based medicine in the field. Despite the numerous studies that have been performed regarding the impact of TBs on diagnosis and treatment, whether cancer guidelines are taken into consideration when recommendations are made—and to what degree—remains an aspect to be investigated.

According to the 2013 Saudi Cancer Registry report,^5^ colorectal cancer is the second most common malignancy in Saudi Arabia. Thus, the purpose of this study was to focus on whether TB decisions adhere to National Comprehensive Cancer Network (NCCN) guidelines, the impact of TBs on patient management, and the compliance of physicians with the TB recommendations.

## METHODS

Our study is a prospective cohort chart review. Data regarding adherence to guidelines, adherence to TB recommendations, and changes made to the management of patients were collected weekly from the TB form. Data collection was conducted for approximately 6 months, from January 2016 to June 2016. All GI cases discussed at the TB during this period were included. Approval was obtained before initiating the study.

### Study Area/Setting

This study was conducted at King Abdulaziz Medical City (Riyadh, Saudi Arabia), which is a tertiary care facility that provides comprehensive cancer care. The GI TB is held on a weekly basis with an average of 20 attendees, including surgeons, medical oncologists, radiation oncologists, pathologists, radiologists, and residents. Three to five GI cancer cases are reviewed per week.

### Study Subjects

All GI cancer cases presented at the GI TB were included.

### Sample Size

All cases presented were included. For this study, approximately 100 cases were expected to be presented at the GI TB.

### Data Collection Methods, Instrument Used, and Measurements

**Fig A1:**
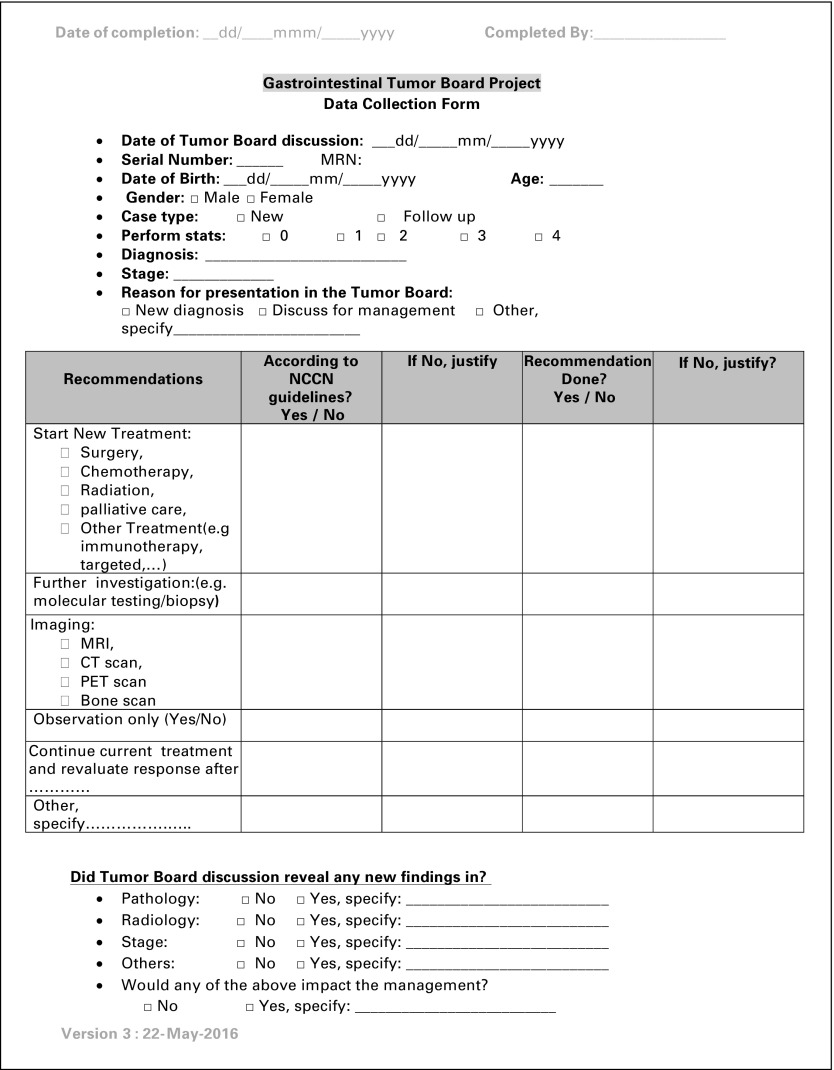
Data collection form.

Data were collected on a weekly basis from patient records and documented in a data collection form (Appendix Figure A1) prepared by the team. The variables measured were change in pathology/radiology/tumor stage, review with a specification, impact on treatment, adherence of recommendations to NCCN guidelines, and implementation of recommendations.

### Data Management and Analysis Plan

Data were described using frequencies and percentages for categorical variables. Data were entered and analyzed using the IBM SPSS Statistics for Macintosh, Version 21.0 (IBM Corp., Armonk, NY).

## RESULTS

All 104 cases presented to the weekly GI TB were included in our study. In 57 cases (55%), the patient’s sex was male, and the median age was 58 (16 to 85) years. Colorectal cancer was the most common diagnosis, comprising 65 cases (63%). The percentages of patients who had stage IV, III, II, and I cancers were 45%, 23%, 10%, and 3%, respectively. Fifty-two cases (50%) were presented to the TB as new cases. The leading reason for presentation to the TB was to discuss the overall management plan, which was accounted for by 82 cases (79%; Appendix Table A1).

Of a total of 158 TB recommendations, 153 (97%) were according to NCCN guidelines, and 138 TB recommendations (87%) were performed within the next 3 months. Starting new treatment was considered in 96 recommendations (69% of all cases). The new treatment most frequently recommended by the TB was chemotherapy (54%). Most of the new treatment recommendations (97%) were according to NCCN guidelines, and 84 recommendations (88%) were done within 3 months after presentation at the GI TB. Justifications for not following the NCCN guidelines included difficult to resect, no tumor response to guideline choice of treatment, patient preference, and physician discretion.

Further investigations were considered in 15 recommendations (14% of all cases). Of all investigations recommended, the most frequent was molecular testing (60%), in nine cases (Appendix Table A2). All 15 recommendations were according to guidelines and were done within 3 months.

Twenty-four recommendations were made for imaging. The most common imaging modality recommended was MRI (37.5%), in nine cases (Appendix Table A2). Of the 24 recommendations, only one (4%) was not according to guidelines. Of all 24 imaging recommendations, 19 (79%) were done within 3 months.

Eight recommendations were considered for observation alone, 50% of which were according to NCCN guidelines, and six cases (75%) were done within the next 3 months. Three recommendations were related to referral to other services. All of them were according to NCCN guidelines and done within the next 3 months. Three recommendations were to continue current treatment and to re-evaluate response after a specific period of time; all of them were according to NCCN guidelines and done within the next 3 months.

During the TB meetings, 31 new findings were unveiled: 11 (11%) pathology, 14 (13%) radiology, four (4%) stage, and two (2%) other. Pathologic findings included the discovery of presence or absence of lymph nodes, increase or decrease in tumor size, changes in the histologic type of cancer, positive or negative margins, lymphovascular and perineural invasion, and risk stratification.

Radiologic findings included the discovery of presence or absence of metastatic deposits, fistulas, masses, perforation, and atrophy. The two findings in the other category were the discovery of a benign cyst and a decision for the patient to undergo surgery.

Of 104 cases, the TB had an impact on the management of 37 cases. The impact was the result of recommendations such as the addition of adjuvant or neoadjuvant chemotherapy or radiotherapy, change of the chemotherapy regimen, discontinuation of chemotherapy, restaging, observation, surgery, transplantation, ultrasound, biopsy, and follow up in the TB.

## DISCUSSION

By analyzing the degree of adherence to NCCN guidelines and following application of the recommendations, this study aimed to measure the effectiveness and quality of care provided by the TB. The first noticeable result was the high rate of adherence by physicians participating in TBs to NCCN guidelines. On the basis of our results, the adherence rate was approximately 97%, which surpasses the results in other published studies (34.5% to 37.2%) that reflect adherence in real-life practice not related to TBs.^6,7^

Another study showed that 84% of all cases presented at the TB were compliant with NCCN guidelines in implementing the treatment plan.^8^ This study and ours suggest that a TB enhances adherence to guidelines as it becomes a reference for discussion and decision making.

The high response of clinicians to TB recommendations is as noteworthy as the value of TB decisions. Ung et al^4^ demonstrated that implementation of the recommendations was completed in 72% of cases, whereas in our study the implementation rate was 87%. For cases in which the recommendations were not followed, the reasons were pre-existing poor patient performance status, declining performance status, clinician preference, the availability of new data, and patient decision.

TB meetings offer physicians the chance to review the cases they have and to reconsider changes in diagnosis and management. A considerable number of management plans (36%) were changed after presentation to the TB. In a study done in the United States, the clinical management was changed in 20% of cases presented at the TB.^2^ The TB changed the management plan in 25% of cases.^9^ The proposed strategies for treating 41% of cases were reformed following the NCCN guidelines.^10^ The TB had a significant effect on changing 40% of treatment plans and 60% of staging and assessment plans.^11^ Another study demonstrated staging modifications or alterations of treatment plans in 60% of patients.^12^ Multidisciplinary teams have had an impact on the management of patients by enhancing the importance of following the NCCN guidelines, which was also shown in more than one-third of cases in our results.

A larger sample size and the inclusion of different tumor boards (other than a GI TB) would increase the strength of our study findings. We also must acknowledge that our findings are derived from one tertiary care center, which may not reflect other health care centers with different resources. In a future study, we would like to determine if measurement of TB performance can be monitored via electronic medical record to capture data related to adherence to recommendations rather than doing it manually.

In conclusion, a multidisciplinary TB enhanced the adherence to guidelines and had an impact on patient management in more than one-third of patients. Among physicians, adherence to recommendations of the TB was high. In addition, future studies should include more than one TB specialty and study the differences between adherence to recommendations and guidelines.

## Figures and Tables

**Table A1:**
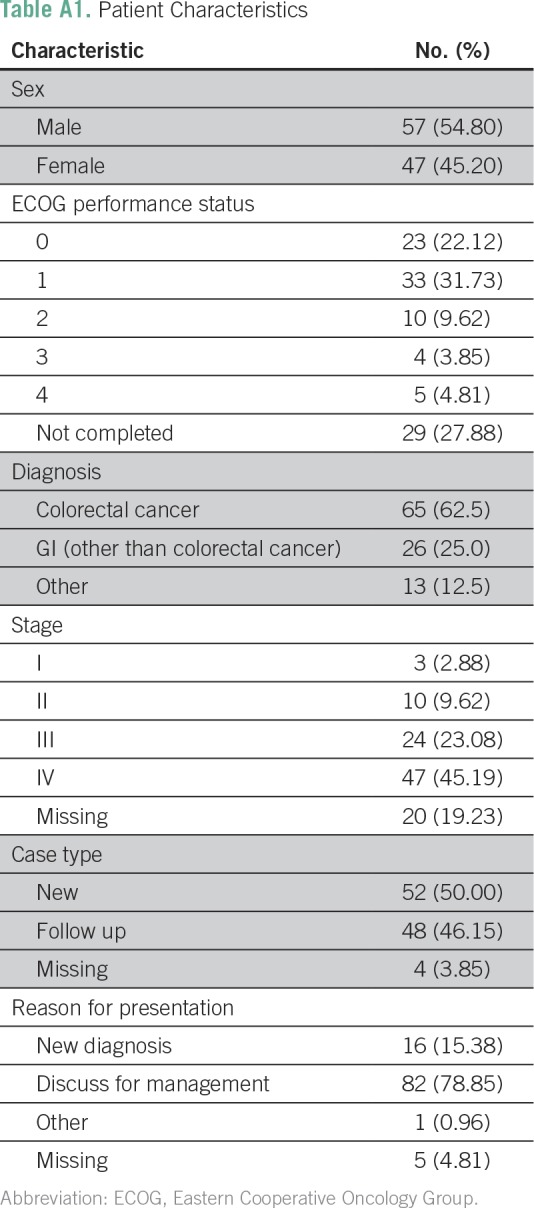
Patient Characteristics

**Table A2:**
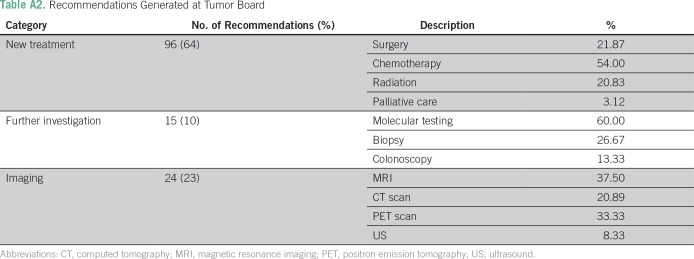
Recommendations Generated at Tumor Board
